# Dilemma of healthcare reform and invention of new discipline of health fiscalogy

**DOI:** 10.1186/s41256-016-0003-x

**Published:** 2016-06-15

**Authors:** Jitong Liu, Jianchun Miao, Dongqi Zhang

**Affiliations:** 1 Institute of Medical Humanities, Peking University Health Science Center, Beijing, China; 2 Peking Union Medical College, Beijing, China; 3 National Medical Examination Center, Beijing, China

**Keywords:** Chinese healthcare reform, Health economics, Health fiscalogy, Governmental responsibility of health

## Abstract

**Background:**

China's Reform and Open up Policy in 1980s has brought rapid economic development to Chinese society. With the deepening of economic reform, the withdrawal of the state in China has had visible and worrisome consequences for health and for the functioning of health services. The new round of healthcare reform after 2009 has made significant achievements on improving fundamental health and bringing back the nature of welfare of health. However, the financing mechanism of health system has not been established, and the underlying reason behind the healthcare reform dilemma and the theoretical solution need to be found.

**Methods:**

This study used the methods of literature review, theoretical research and comparative research to summarize and analyze the reasons and solutions of current dilemma in healthcare reform, and created the new discipline of health fiscalogy through theoretical analysis and vertical and horizontal comparison of healthcare system, especially health financing.

**Results:**

Dilemma in healthcare system emerged from the circumstances of rapid process of industrialization, urbanization and population aging, including the profit-driven phenomena, tendency of excessive marketization in public hospitals, strained doctor-patient relationship, high disease burden on individuals and families, and so on. It can be concluded that the theoretical basis of healthcare system and the nature of health resources are crucial in solving the dilemma of healthcare reform. The theoretical basis of healthcare reform should be health fiscalogy focusing on government as the main body of health care responsibility rather than health economics focusing on anti-monopoly. There are two key differences between health economics and health fiscalogy: responsible person/department of disease and health welfare, and nature of resource. The new discipline of health fiscalogy has universal and important implications on both China’s healthcare reform and the healthcare reform in the world.

**Conclusions:**

China’s healthcare reform should return from the paradigm of health economics and marketization financing model to the paradigm of health fiscalogy and government-led financing model, which is reflected in the main position of government and social welfare.

## Background

China's Reform and Open up Policy in 1980s has brought rapid economic development to Chinese society, however, with the deepening of economic reform, the withdrawal of the state in China has had visible and worrisome consequences for health and for the functioning of health services [32]. One of the main consequences is that health institutions has become more and more profit-driven under the circumstances of decreasing input of government and increasing needs for health services [14].

After SARS in 2003, Chinese government proposed to make further research on healthcare reform, and "Opinion of the CPC Central Committee and the State Council on Deepening the Healthcare reform" has been issued in 2009 to make health quality back to reform agenda. The new round of healthcare reform has made significant achievements on improving fundamental health and bringing back the nature of welfare of health [12]. However, the financing mechanism of health system has not been established and the profit-driven phenomena still exist [12]. People are still complaining about the poor access and high fee in health services and cases in which doctors are injured or killed happened more and more frequently [15]. A national investigation on hospital violence during 2003 to 2012 in China's public hospitals showed that the percentage of medical workers who had experienced physical attack and injured from the violence increased from 47.7 % in 2008 to 63.7 % in 2012 [9]. The strained doctor-patient relationship has become an undesirable phenominon in healthcare reform.

This paper will focus on the financing mechanism of health system and explain the underlying reason behind the healthcare reform dilemma and bring up the theoretical solution in healthcare reform in China.

## Methods

Literature review, theoretical research and comparative research methods were used in summarizing and analyzing the reasons and solutions of current dilemma in healthcare reform [1, 2]. The authors reviewed the mainstream opinions on reasons for dilemma in healthcare reform, made theoretical analysis from perspectives of the responsible person/department of disease and the nature of health expenditure, and made vertical comparison between medical situations in recent years and before 1980s and horizontal comparison between Chinese situation and abroad. With the above research, authors put forward the new disciplinary view of health fiscalogy which may be the key point to solve the dilemma in current Chinese healthcare reform.

## Results

### Definition of health financing and health economics

As indicated in World Health Report 2010, health financing is much more than a matter of raising money for health. It is also a matter of who is asked to pay, when they pay, and how the money raised is spent. Resources can be collected through general or specific taxation; compulsory or voluntary health insurance contributions; direct out-of-pocket payments; and donations [33].

Health economics belongs to the discipline of economics, with the core problem of how to distribute resources in an efficient way. It mainly focuses on the influences of health on economic and social development; and the allocation of health resources to achieve best wellbeing [31].

### Dilemma in healthcare reform

After 1985, especially after 2000, the phenomena of increasing income from medical services in public hospitals became more and more obvious, while the direct expenditure from government decreased sharply, from 35 % in mid 1980s to less than 10 % in 2012, making the economic burden for patients increased [11]. Personal cash payment peaked as the main part of health expenditure in 2000 [20]. The direct result is high disease burden on individuals and families. The "Opinion of the CPC Central Committee and the State Council on Deepening the Healthcare reform" issued in 2009 has put "establishment and improvement of basic healthcare system" as the overall objective. With concerted efforts from Chinese government and relevant stakeholders, healthcare reform after 2009 has achieved great progress, including the increasing fiscal expenditure on healthcare system (increased by 9.62 % in 2014 than in 2013), decreasing percentage of direct out-of-pocket payments (decreased by 10 % than before healthcare reform), and improved health insurance coverage and quality [6].

However, with the rapid process of industrialization, urbanization and population aging, as well as the "new normal" of economic development, the conflicts between downturn of economy, lack of health resources and the increasing health needs of the public are becoming more and more obvious [6]. Dilemma emerged from these conflicts, leading to the deterioration of medical environment.

According to the “Report of Hurting Doctors with Violence in Hospitals” by Chinese Hospital Association in 2013, the cases of hurting doctors with violence in hospitals increased year by year, from 20.6 cases per hospital per year in 2008 to 27.3 in 2012. There are many reasons behind this situation, including high expenditure in health, lack of medical knowledge of patients, excessive hospitalization and medical care, malfunction of referral mechanism, misleading media publicity, and so on [13]. But the fundamental reason is the direct economic conflict between doctors and patients. The financing mechanism of health system and proper personnel salary system have not been established, which directly resulted in the existing of profit-driven phenomena. Public hospitals in China have shown the tendency of excessive marketization [5]. As the result, the image of hospitals has been critically damaged by its pursuing economic interest [10]; the primary health care and public healthcare are marginalized due to the relatively lower capability of making profit; the “professional agency relationship” between doctors and patients was replaced by direct economic interest.

### Theoretical and comparative analysis

Currently, there are multiple diagnostic explanations on this dilemma. The mainstream voice is from economists. They think the monopoly of Chinese government should be blamed, and suggest that if government open the health market, there would be no problem [37]. Health institutions say that the main reason is the gap between health needs and health services providing capability, and they focus on how to identify the function of government in health sector [8]. However the functional departments of the State Council think that the National Health and Family Planning Commission (former Ministry of Health) is in charge of both running hospitals and authorizing them, so the systematic problem of “implementation not separated with authorization” should account for the practical troubles [17]. However, all of those explanations ignored the fundamental key point of healthcare reform, which is the nature of health services and health care system. The essence of “welfare” is to provide “de-commodification” service but not “commodification” service or profitable service.

Comparing with China's healthcare system before 1980s, when public hospitals mainly relied on fiscal allocation and healthcare institutional network was basically established to cover the urban and rural areas [14], healthcare system after 1980s has turned the theoretical basis of healthcare from “social welfare” to “health economics” under the macro background of economic system reform and state-owned enterprises reform, and the influence of main stream social value of “the first priority should be efficiency, with due consideration given to equity”. The “Decision of the Central Committee of the Communist Party of China and the State Council Concerning Health Reform and Development” in 1997 stated that “the nature of health service in our country is philanthropy while the government pursues a certain welfare policy”. Since then, the nature of health care changed from unitary social welfare to binary nature of welfare and philanthropy [29]. China’s medical institutions have been strengthening economic management, implementing the contract responsibility system in hospital departments, and opening medical market for higher service income, thus the model of marketization of financing in hospitals gradually exceeded the model of governmental welfare responsibility and public finance for health care [26]. In 1991, “Network for Training and Research on Health Economics and Financing” was jointly established by the World Bank Economic Development Institute and China’s National Health and Family Planning Commission, rapidly introducing “Americanized” health economics into China’s economics and health system [31]. The direction of healthcare reform changed from “health equity and general health welfare” to “improvement of economic efficiency of health services”. In short, the paradigm of health economics defeated the thought of health welfare after 1990s, and became the values and theoretical basis of healthcare reform in china.

**Fig. 1:**
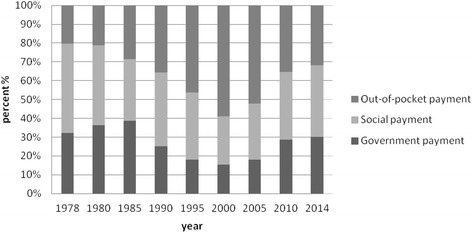
Change of component and proportion of health expenditure from 1978 to 2014. This figure is produced by authors according to data from Chinese Health Statistics Yearbook, Beijing, Beijing Union Medical University Press, 2015

The typical health financing resources are general tax or social security tax in most western countries. For instance, countries with general tax as the main health financing resources include the UK and some Northern European countries, such as Sweden, Finland and Norway; countries with social insurance as the main health financing resources include Germany, France, and Belgium [35]. While in China, direct out-of-pocket payment cannot be ignored in health financing, especially before the new round of healthcare reform in 2009. Figure 1 shows the trend of structural change in health expenditure since China’s Reform and Open up Policy. It can be concluded from the figure that the out-of-pocket payment on health in China peaked in 2000, and decreased gradually to 32 % in 2014. A survey in eighty-nine countries covering 89 percent of the world's population suggests that financial catastrophe in households is positively correlated with the relative importance of out-of-pocket payments in total health spending [36]. Therefore, we could anticipate even better wellbeing for people's livelihood in China if direct out-of-pocket payment continues to decrease and government takes the leading responsibility of health financing, making healthcare returns to the nature of "welfare" again.

From the above vertical comparison between medical situations in recent years and before 1980s and horizontal comparison between Chinese situation and abroad, it can be concluded that the theoretical basis of healthcare system and the nature of health resources are crucial in solving the dilemma of healthcare reform.

### Creation of health fiscalogy

Health fiscalogy is one of China’s great contributions to public fiscal system and healthcare system. It comes from the practices and lessons of decades of healthcare reform in China [18]. Literature review indicates that there are no articles or the concept of health fiscalogy in western welfare countries except for the U.S. where there are articles on health fiscal administration [3]. Economics in Chinese refers particularly to activities based on market. As a neutral word, “finance” is used to indicate the channels and actions of raising fund, but it is not related with responsibility of government. There is no accurate word to express the connotation of Chinese word “Cai Zheng (财政)” such as governmental will, political commitment, social welfare and so on [34]. Therefore, the authors prefer to use a new saying “health fiscalogy” to express health finance in Chinese meaning, and distinguish it with “health economics”. The disciplinary view and theoretical basis of the two are totally different.

The nature of health services and health care system should be “social welfare”, which decide who should take the responsibility of health financing [19]. Therefore, the issue of theoretical basis of healthcare reform comes into being. Whether it should be health economics focusing on anti-monopoly, or health fiscalogy focusing on government as the main body of health care responsibility will have direct influence on the health system building, general design of healthcare reform plan, and direction of healthcare reform.

### Essential differences between health economics and health fiscalogy

Generally speaking, the most important and key differences of health economics and health fiscalogy are from the following two perspectives:

Firstly, who should be responsible of disease and health welfare? The view of economics and health economics is that disease is personal trouble, and patients and their family should be responsible for his/her health status and pay for themselves. In the contrary, the view of health fiscalogy is that disease is not pure personal trouble, but partly due to complicated social factors. The determinants of health status of individuals and people include social factors, therefore society especially government should take the main responsibility of its people’s health [38]. Actually, the distribution of health social responsibility mainly addresses “who should pay for the disease”, that is who is responsible for the disease. This means health fiscalogy concerns about responsibility of government in health care, while health economics concerns about individual responsibility.

Secondly, what is the nature of money and resource? Economics and health economics concern about business capital, market financing, and market mechanism, and the nature of money and resource is business. In the contrary, fiscalogy and health fiscalogy concern about public finance and tax, including philanthropy and other non-profit social fund, and the nature of money and resource is public resource and public welfare, reflecting the welfare responsibility of government and general social public benefit [25]. Although the combination of public good and private provision, that is money and resource with nature of public finance are provided through business or market, could make some misunderstanding [28], their welfare nature will not change.

Table 1 summarizes the main differences between health fiscalogy and health economics.

**Table 1:** Substantial differences between health fiscalogy and health economics

Items	Health economics	Health fiscalogy
Historical origin	Historical product of modern society	Concomitant of society with long history
Discipline	Branch discipline of economics	Public economics and public finance
Nature of policy	Policy of economics and market	Public policy and social policy
Objective of policy	Economic efficiency and making profit	Social equity and health equity
Disciplinary level	Micro and mid level	Macro and strategic
Theoretical basis	Economics and market competition	Governmental responsibility and social rights
View of research	Resource allocation and market distribution	Resource allocation and government distribution
Nature of questions	Economic questions	Political and social questions
Research topics	Market mechanism and economic efficiency	Governmental responsibility and social equity
Behavior subjects	Market sector	Government, Non-profit organizations
Research subjects	Market economy and relationship between supply and need	Governmental decision and public policy
Analysis unit	Individual behavior and preference	Collective behavior and public choice
Research scope	Whole process of manufacturing, transportation and consumption	Income, budget, implementation, supervision
Role of government	Insignificant and secondary	Dominant and key
Role of market	Core and decisive	Insignificant and secondary
The third sector	Civil society and philanthropy	Important factors and other supplementary roles
Research method	Model and econometrics	Public policy analysis and comparison
Research team	Mainly economists	Experts on politics and public management
Function	Economic analysis and model building	Public finance and budget management
Effects	Economic efficiency and economic welfare	Social equity and health equity

### Universal implication of health fiscalogy

The invention of the new discipline of health fiscalogy has universal and important implications on both China’s healthcare reform and the healthcare reform in the world.

Firstly, the nature of healthcare systems is social welfare, an important part of “de-commodification” social service, which is the foundation to identify the nature of disease burden as well as the social division of health care responsibility [16]. The health professional trust and system trust are also based on people’s social rights, health equity and people-oriented medical services in the nature of welfare. Modern medical services and medical professionalism should not be related with market mechanism, commodity services and economic benefit at all [23].

Secondly, health financing model and compensatory policy of medical institutions are the most fundamental and important issue in health care system. We can learn from China’s healthcare reform that health financing is the core issue in healthcare reform. During the establishment and improvement of health care system and public health service system, a stable financing mechanism by government is indispensable for both consumers and providers [30]. Undoubtedly, the view of health fiscalogy contributes to health financing system reform in providing a correct theoretical basis, pointing out the development direction of deepening healthcare reform. Therefore, the policy objective and strategic objective of China’s healthcare reform practice should be the change of health financing model to government-led modern health financing model with Chinese characteristics [21].

Development of healthcare reform in western countries and the dilemma in China’s healthcare reform provide important lessons and political implications for China and other countries, help in strengthening health wellbeing, and illustrate that the essence of health fiscalogy is governmental responsibility of social welfare and universal health care. Only if Chinese government takes the main responsibility of universal health care and a health fiscal system and financing model with Chinese characteristics are built, can China’s image as “economic country” be changed to “welfare state”, the harmonious doctor-patient relationship be established, people’s health status be improved both physically and mentally, and the whole society’s health and wellbeing be maximized [22].

Thirdly, health fiscalogy is an important branch of public finance. The disciplinary building and theoretical research of health fiscalogy will certainly enrich and develop the theory and practice of public finance, and provide a fresh example for theoretical fiscalogy and public finance [7].

Fourthly, the global health care system is gradually formed, requiring more and deeper international cooperation in health arena, including healthcare reform and health financing model [4]. The new discipline of health fiscalogy provides a theoretical, policy and disciplinary basis for global health fiscalogy.

## Discussion and Conclusions

China’s healthcare reform has encountered a dilemma because of the misunderstanding of the nature, function and direction of healthcare system. The deeper reason in theory is that people are influenced by the thought of economics and health economics. Health institutions are regarded as independent economic entities by introducing market mechanism and factors, which is the main reason for the dilemma in healthcare reform, including dissatisfactory doctor-patient relationship. This brings us the questions such as what are the objectives of China’s development, the economic system reform, and the healthcare reform. Therefore, China’s healthcare reform should be led by correct values and theories, change the theoretical and value basis, identify the main responsibility of government in healthcare system, and return from the paradigm of health economics and marketization financing model to the paradigm of health fiscalogy and government-led financing model. The institutional return is not just a historical repeat, but to build the public fiscal system in a higher level.

More than thirty years’ healthcare reform practice proves that the paradigm of health economics and marketization of financing model have brought negative influences to society, which, to some extent, created new social risks, aggravated social conflicts, and damaged the social trust between doctors and patients. The “professional agency relationship” between doctors and patients was replaced by direct economic interest, further promoting the strained doctor-patient relationship. Historical experience from health care systems development around the world shows that health service is a high-risk and high-uncertainty industry based on people’s value of equity and welfare which needs the social trust, professional trust and institutional trust in social welfare system the most, but not the market system inspiring the ugly and greedy side of human beings [27]. Health economics and health fiscalogy are the two theories with essential distinctions, in which the market and government are different in nature. Government takes the main responsibility in health fiscalogy.

Encouragingly, it has become the common sense of the whole society and also the direction of governmental functional change to build a harmonious society and welfare system with Chinese characteristics, to improve people’s health and welfare by changing the pattern of economic development, coordinating urban and rural development, building a people-oriented well-off society, and sharing the reform achievements among people. The welfare nature of health care system is the general rule and international convention of many countries and also the theoretical basis for the disciplinary view of health fiscalogy. The year of 2010 is the first year of China’s social welfare, child welfare, the disabled welfare, the elderly welfare, family welfare and patient welfare, marking the era of social policy, social legislation and social welfare in China, and the change of governmental responsibility and health financing model [24]. The establishment of health fiscal system based on health fiscalogy is the core of healthcare reform and development around the world. The healthcare reform practices from China and other western countries illustrate that the health fiscalogy has universal significance on healthcare reform and development.

Currently, the top priority is to form the “social welfare consensus”, and to develop and improve the health fiscal system framework with Chinese characteristics with joint efforts. The social significance of the establishment of health fiscalogy and health fiscal system framework in China will be comprehensive, systematic, profound, and universal.
